# What happens to intimate partner violence studies registered on clinicaltrials.gov? A systematic review of a clinical trials registry

**DOI:** 10.1186/s13063-019-3412-6

**Published:** 2019-05-27

**Authors:** Kim Madden, Kerry Tai, Patricia Schneider, Nathan Evaniew, Michelle A. Ghert, Mohit Bhandari

**Affiliations:** 1 Division of Orthopaedic Surgery, Department of Surgery, McMaster University, Hamilton, ON Canada; 2 Research Institute of St. Joe’s Hamilton, Hamilton, ON Canada; 3 Department of Health Research Methods, Evidence, and Impact, McMaster University, Hamilton, ON Canada

**Keywords:** Intimate partner violence, Systematic review, Trial registration, Methodology, Publication bias

## Abstract

**Background:**

There is an increasing number of interventions aimed at reducing the incidence and improving the identification and management of intimate partner violence (IPV), which are being tested in randomized clinical trials. Publication bias, improper reporting, and selective reporting in clinical trials have led to widespread adoption of pre-registration of clinical trials. Non-publication of study results leads to inefficiency, ethical issues, and scientific issues with the IPV literature. When study results and methodology are not made available through publication or other public means, the results cannot be used to their full potential. The objective of this study was to determine the publication rates of IPV trials registered in a large clinical trial registry.

**Methods:**

We conducted a systematic review of all IPV-related clinicaltrials.gov records and determined whether the studies that had been completed for ≥ 18 months have been published in a peer-reviewed journal or in the clinicaltrials.gov registry. Two authors extensively searched the literature and contacted study investigators to locate full-text publications for each included study.

**Results:**

Of 83 completed IPV-related trials registered on clinicaltrials.gov, 64 (77.1%, 95% CI: 66.6–85.6) were subsequently published in full-text form. Of the 19 unpublished studies, authors confirmed that there was no publication for 11 studies; we were unable to contact the investigator or locate a publication for the remaining eight studies. Only four studies (all published) posted their results on clinicaltrials.gov upon completion.

**Conclusion:**

Approximately one in four IPV trials are not published 18 months after completion, indicating that clinicians, researchers, and other evidence users should consider whether publication bias might affect their interpretation of the IPV literature. Further research is warranted to understand reasons for non-publication of IPV research and methods to improve publication rates.

## Background

Intimate partner violence (IPV), also known as spouse abuse and domestic violence, affects one in three women globally [1]. IPV is an important social issue that has well-documented health implications, including poor mental health [2] musculoskeletal injuries [3, 4] reduced quality of life [5], and even death in severe cases [6]. There is a growing number of interventions in healthcare settings for victims of IPV; these interventions are increasingly being evaluated by clinical trials [7, 8]. As the literature on IPV interventions grows, it is important to ensure transparency of study design and accurate trial reporting, and to evaluate potential bias in the literature, so that evidence users are not misled by inaccurate or inappropriate reporting. Additionally, since the effectiveness of IPV interventions is often highly controversial [9–11], it is important to have as much high-quality published evidence as possible.

It is important to register clinical trials for many reasons, including ethical obligations, legal obligations, and scientific considerations. Registering clinical trials allows patients and research participants to access information about clinical trials in which they could potentially participate (the registry’s original purpose) [12]. Granting agencies and investigators can search trial registries to determine if there are any ongoing studies that might make a planned study redundant [12]. This usage aims to improve efficiency of clinical research and allocation of funding. Trial registries are also important for study methodology. Prospectively registering a study aims to reduce publication bias, selective reporting bias, and improve transparency [12]. Trial registries are publicly available databases, making it easy to find all trials that have been initiated for a particular intervention of interest. It is this transparency that should encourage investigators to publish their results regardless of whether they are positive, negative, or inconclusive, which has the potential to limit publication bias [13]. Because trial registry is required to occur before enrollment of the first patient, one can see in the trial record the originally planned eligibility criteria, intervention, comparison group, outcomes, and other important elements of the protocol. This means that registry records can be used to determine if the study plan changed over time so that the reader can assess if there is a risk of bias from selective reporting. Investigators are not currently required to register other study designs like observational studies, but it is encouraged.

Previous studies have reported very low rates of publication among studies registered on clincialtrials.gov and other trial registries. Ohnmeiss [14] found that only 38.9% of registered spine trials were published. Similarly, 22.8% of arthroplasty trials [15], 43.2% of trauma trials [16], 54% of macular degeneration trials [17], 54% of diagnostic accuracy studies [18], and 58.8% of sports medicine trials [19] are published. No previous studies have reported on the publication rates of registered studies in the IPV field. The current study can shed light on the current state of the IPV literature in terms of publication rates and potential for publication bias.

We conducted a systematic review of IPV studies registered with clinicaltrials.gov with the objective of determining the proportion of studies that have been published within 18 months of the trial being reported as complete on clinicaltrials.gov. Additionally, we aimed to explore the characteristics of trials that are published versus those that are not published.

## Methods

### Identification of registry records

We performed a search of the clinicaltrials.gov trial registry on 12 September 2017 using the terms “spouse abuse” OR “domestic violence” OR “partner violence” OR “partner abuse”. Two authors (KM and KT) independently reviewed all study titles, outcomes, interventions, and conditions that the search identified. Studies were excluded if they focused only on child abuse, or if the title, outcomes, interventions, and conditions did not mention intimate partner violence or a related term such as domestic violence. We included all study designs (e.g. randomized trials, non-randomized studies, prospective cohort studies).

Once the relevant studies were identified, we determined whether the studies were “completed” or “not yet complete” based on what was reported in the clinicaltrials.gov record. At this point we excluded studies that were listed as “terminated,” “withdrawn,” or “suspended” in the registry. Additionally, we excluded studies with a date of completion in the past 18 months, in order to account for a reasonable time delay between trial completion and publication. We chose 18 months as our cut-off to allow sufficient time after the end of enrollment for data cleaning, data analysis, and manuscript writing, plus several months for review by a journal and subsequent publication. The World Health Organization (WHO) recommends publication within 12 months of study completion, but up to 24 months may be allowable [20]. Previous studies of publication rates of registered studies have used a cut-off of 18 months [14].

### Identification of publications

We searched for each publication in AMED (Allied and Complementary Medicine Database), Embase, Global Health, Healthstar, Medline, and PsycInfo using the Ovid search interface, plus Google Scholar. We searched the clinicaltrials.gov trial identification number first; then, if the publication could not be found, we searched the publication databases using the principal investigator’s (PI) last name plus trial keywords. An additional author (KT and PS) attempted to find the publications that the first author (KM) could not locate. We also attempted, on up to three occasions, to contact the PI listed on the clinicaltrials.gov record for publications that could not be located and for publications where we were unsure if they matched the clinicaltrials.gov record. We defined “publication” as a paper published in a peer-reviewed journal (i.e. not an internal report to industry, funding agency, or government). In addition, the publication had to contain results to be considered complete (protocol papers and initial reports were excluded).

### Data collection

We exported the results of the clinicaltrials.gov search into a study database. For each study with a corresponding publication, one author (KM) extracted the month and year of publication, country, study design, intervention(s), funding source, and whether the authors reported the trial registry number. A second author (KT) verified all data points. Disagreements were settled by consensus or by consulting the senior author (MB).

### Data analysis

We calculated agreement for inclusion using the kappa statistic with 95% confidence interval (CI) using the GraphPad kappa calculator (http://graphpad.com/quickcalcs/kappa2/). We used SPSS version 24 to conduct Fisher’s exact tests and t-tests comparing unpublished and published study characteristics, and to construct a Kaplan–Meier survival curve for publication status (with an “event” defined as publication) and reported the median survival time with 95% CI. We present descriptive statistics using frequencies and percentages, as appropriate. We also conducted a sensitivity analysis using a cut-off of 24 months since completion, per the upper limit of the WHO’s recommendations for making study results available. We conducted an exploratory multivariable binary logistic regression to determine if country, study design, and funding source were associated with publication.

## Results

### Search results

**Fig. 1:**
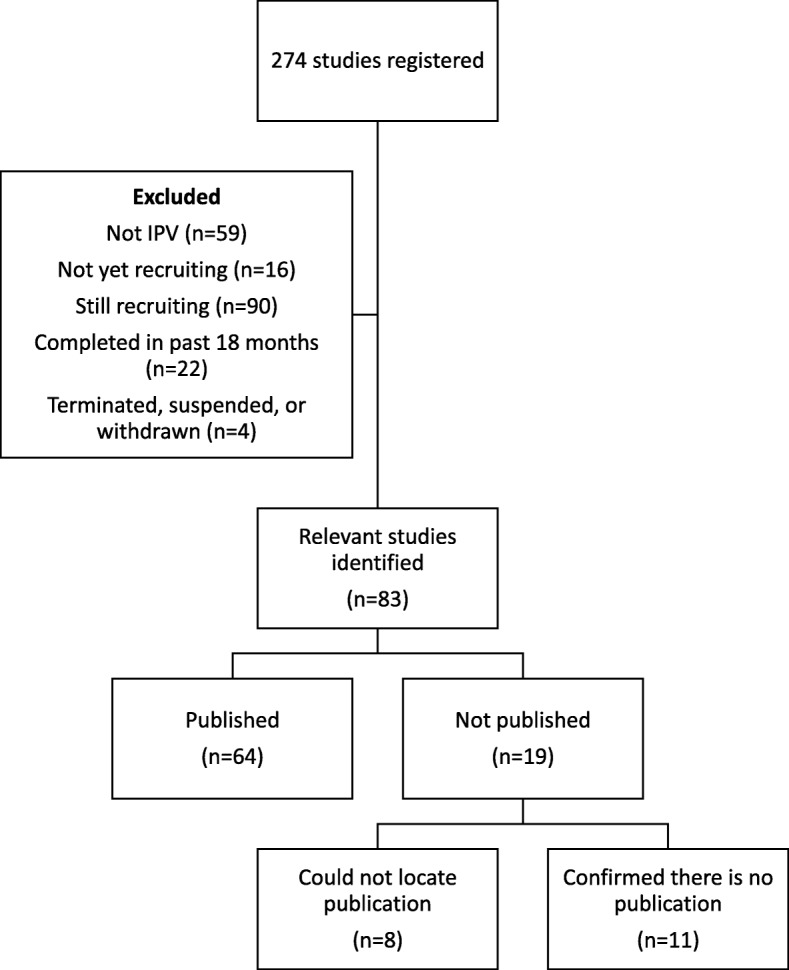
Study flow *diagram*

We identified 274 study records in clinicaltrials.gov (Fig. 1). We excluded 59 of these studies because they did not relate to IPV and 106 because they were not yet completed. Four studies were withdrawn, suspended, or terminated; 22 had been completed < 18 months before the registry search. Thus, there were 83 relevant clinicaltrials.gov records for which we sought matching publications. Inter-observer agreement for inclusion was almost perfect (kappa = 0.97, 95% CI: 0.93–1.00).

### Published registered studies

**Fig. 2:**
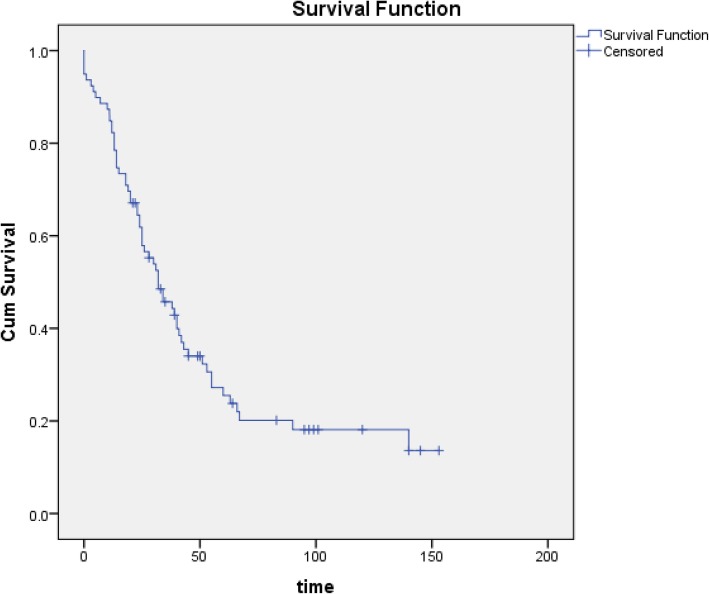
Kaplan–Meier survival curve for time to publication

Of the 83 studies for which we sought full-text publications, we were able to locate 64 (77.1%, 95% CI: 66.6–85.6). Of the remaining 19, authors of 11 studies confirmed that there is no publication; we were unable to contact the PI or to locate the publication for eight studies. Reasons given by authors for not having a published paper included that the publication is still in preparation or review, the results were uninteresting (i.e. negative), the study had methodological flaws, and the study was part of a PhD dissertation and was never published. Median time to publication was 32.0 months (95% CI: 21.8–42.2) (Fig. 2). Using a cut-off of 24 months since study completion, 60/77 studies were published (77.9%).

### Study characteristics

Study characteristics for published and unpublished studies are shown in Table 1. Most studies were from the United States (52/83, 62.7%) and were randomized controlled trials (RCTs) (66/83, 79.5%).

**Table 1:** Study characteristics

Study characteristic	Published studies (*N* = 64)	Unpublished studies (*N* = 19)
*Study design*		
RCT	52	14
Non-RCT	12	5
*Country*		
USA	43	9
Other	21	10
*Funding type*		
Government	43	11
Foundation/Association	7	0
Industry	1	0
Unclear/Not reported	13	8
*Results reported in registry*		
Yes	4	0
No	60	19
*NCT number reported in publication*		
Yes	38	
No	26	

Few studies (4/83, 4.8%) posted their results to clinicaltrials.gov. Interestingly, only 38/64 published studies (59.4%) reported their clinicaltrials.gov registration number in the published paper, despite that reporting the registration number is required by CONSORT guidelines [21]. We did not find any evidence that study design (RCT vs non-RCT; OR: 1.67, 95% CI: 0.48–5.86) or country (USA vs non-USA; OR: 2.23, 95% CI: 0.77–6.50) or funding source (Government/Non-Profit/Industry vs Unreported; OR: 2.817, 95% CI: 0.92–8.64) were associated with publication; however, with a small sample size, these results should be interpreted with caution.

## Discussion

Clinicaltrials.gov and other trial registries are important tools to aid in transparency of conducting and reporting clinical research and reducing bias associated with non-publication. Since IPV interventions and associated trials are a growing area of interest for clinicians and knowledge users, it is important to critically evaluate the quality of this body of literature in order to make informed decisions. This systematic review of clinicaltrials.gov records found that nearly one in four IPV-related studies are not published at 18 months or longer after being reported as completed on clinicaltrials.gov. The non-publication rate was nearly the same (22.1%) when using a cut-off of 24 months instead of 18 months. There was no evidence that study design, country, or funding source are predictive of publication, but this finding should be interpreted with caution due to small numbers.

Publication bias is a well-documented phenomenon that arises when negative studies are not published and only positive studies are available to users of medical literature and systematic reviewers [22]. The effect is that interventions appear to be more effective than they actually are, thereby misleading clinicians and others seeking to apply results to clinical practice [22]. Some of the investigators contacted for the current review stated that they did not publish their study because they perceived that the study was not impactful (i.e. negative results), indicating the presence of publication bias. The most common reason for non-publication given by authors was that the paper was still in review at a journal. Although negative trials have similar [23] or better [24] methodological quality compared to positive trials, it often takes significantly longer for negative trials to be published compared to positive trials [25]. However, there is evidence that much of the decision not to publish negative trials is made by the author as opposed to journal editors in top medical journals [26]; therefore, authors must be aware of the consequences of publication bias and make all reasonable efforts to publish studies regardless of perceived impact or statistical significance.

Although there are other methods of making results of trials available, publication of study results in a peer-reviewed journal is the classic method of disseminating results to those who can use the knowledge in practice and in future research. Many other methods of dissemination are not publicly available except to a very select group of people (e.g. conference presentations, internal policy documents). Additionally, the full peer-reviewed publication usually contains the most comprehensive description of the study, allowing for proper critical appraisal and inclusion in knowledge syntheses. Since effective knowledge translation and exchange should an important goal of health research, by failing to publish studies, research funding is not used to its fullest potential.

Previous studies have reported very low publication rates in other fields. Ross et al. [27] randomly sampled 10% of all trials in a trials registry and found a publication rate of < 50%. Similarly, with conference presentations, only 49% of poster and podium presentations in orthopedic surgery were published five years after presentation at the American Academy of Orthopedic Surgeons and 64% after presentation at the Orthopedic Trauma Association [28, 29]. It is also possible that other factors affect publication rate. For example, Hakala et al. [30] found that “stalled drugs” (i.e. drugs that reached late stage testing but were discontinued) had a publication rate of only 37% compared with licensed drugs that had a publication rate of 75%. It is unclear whether there is a real difference between IPV research and other fields with respect to publication rates or if comparisons with other similar reviews are limited by differing methodologies.

A strength of this review is our exhaustive attempts to locate published studies using multiple techniques and multiple attempts to contact study investigators. Previous similar studies (e.g. [14, 19]) rarely attempted to contact investigators. This study has a few limitations as well. It is possible that some of the eight studies for which we were unable to locate a publication were actually published. However, the systematic and thorough design of this review with comprehensive searching, double-checking, and contact with investigators attempts to minimize this possibility. Current recommendations for systematic reviews suggest searching Medline, Embase, and the Cochrane Register at minimum [31]. We exceeded this minimum recommendation in our search strategy, enhancing the strength of our conclusions. It is also a possibility that some of the eight studies that we were unable to locate were published in gray literature or journals that are not indexed in major databases, but our conclusions would remain the same, since such publications would not be easily accessible by a general user of medical literature.

We were unable to determine the association between industry funding and non-publication due to small numbers. Future research could investigate the impact of industry funding on IPV studies. We were unable to determine whether statistical significance (i.e. a positive versus negative trial) was related to non-publication because it is not possible to determine the statistical significance of unpublished studies, so we cannot make comparisons between published and unpublished studies. We did not examine the quality of the literature because the primary outcome was non-publication. It is not possible to evaluate the quality of studies that are not published. Additionally, we were able to gather only limited data on reasons why studies are not published in IPV-related research as it was outside the scope of this study; however, it warrants further research. There may be reasons unique to IPV research why studies are not published. For example, members of the current study team experienced rejection of a publication when we attempted to publish in a specialized surgery journal because the editor did not believe that IPV is a surgeon’s issue.

## Conclusions

Approximately one in four registered IPV studies are not published following completion, which means that clinicians, researchers, and other evidence users should consider whether publication bias might affect their interpretation of the IPV literature. Publication bias in IPV literature could lead to an over-estimation of the effectiveness of IPV interventions which could mislead clinicians and policymakers. Additionally, the non-publication of completed IPV studies indicates that research funding is wasted. Further research is warranted to understand reasons for non-publication of IPV research and methods to improve publication rates. Investigators of completed studies as well as journal editors should be aware of the consequences of publication bias.

## Data Availability

The datasets used and/or analyzed during the current study are available from the corresponding author on reasonable request.

## Abbreviations

AMED: Allied and Complementary Medicine Database
CI: Confidence interval
CIHR: Canadian Institutes of Health Research
CONSORT: Consolidated Standards of Reporting Trials
FDA: Food and Drug Administration
FDAAA: Food and Drug Administration Amendments Act
GRADE: Grading of Recommendations Assessment, Development and Evaluation
ICMJE: International Committee of Medical Journal Editors
IPV: Intimate partner violence
LMIC: Low- and middle-income countries
NIH: National Institutes of Health
OR: Odds ratio
PI: Principal investigator
RCT: Randomized controlled trial
WHO: World Health Organization
